# A Numerical Study on Structural Performance of Railway Sleepers Using Ultra High-Performance Concrete (UHPC)

**DOI:** 10.3390/ma14112979

**Published:** 2021-05-31

**Authors:** Moochul Shin, Younghoon Bae, Sukhoon Pyo

**Affiliations:** 1 Department of Civil and Environmental Engineering, Western New England University, Springfield, MA 01119, USA; moochul.shin@wne.edu; 2 Korea Railroad Research Institute, 176 Chuldobangmulgwan-ro, Uiwang-si 16105, Korea; 3 Department of Urban and Environmental Engineering, Ulsan National Institute of Science and Technology (UNIST), Ulsan 44919, Korea

**Keywords:** ultra high-performance concrete (UHPC), railway sleeper, static bending test, numerical simulation, structural performance

## Abstract

This numerical study investigates the structural performance of railway sleepers made of ultra high-performance concrete (UHPC). First, numerical concrete sleepers are developed, and the tensile stress-strain relationship obtained from the direct tension test on the UHPC coupons is used for the tensile constitutive model after applying a fiber orientation reduction factor. The numerical sleeper models are validated with the experimental data in terms of the force and crack-width relationship. Second, using the developed models, a parametric study is performed to investigate the performance of the UHPC sleepers while considering various design/mechanical/geometrical parameters: steel fiber contents, size of the cross-section, and diameter and strength of prestressing (PS) tendons. The simulation results indicate that the size of the cross-section has the most impacts on the performance, while the effect of yielding strengths of PS tendons is minimal among all the parameters. Engineers need to pay attention to efficiency and an economical factor when using a larger cross-section, since sleepers with larger cross-sections can be an over-designed sleeper. This study suggests an economical design factor for engineers to evaluate what combination of parameters would be economical designs.

## 1. Introduction

In a railway track structure system, sleepers (or ties) perform critical functions by transferring and distributing train loadings from rail to ballast or concrete slab. The critical components undergo repeated train loading and impact loading; however, the exact load transfer mechanism within the sleeper is still unclear due to uneven ballast support conditions and irregular surface conditions of rail and wheels. Due to inaccurately identified loads and support conditions, various parts of sleepers can have damages such as center-binding crack, and flexural and/or shear cracks at the rail-seat section. Nowadays, the railway industry has paid more attention to how to improve the service life of sleepers not only because of increasing axle loads, speed, and traffic volume, but also because of increasing maintenance costs including expensive sleeper replacing costs [1]. 

Concrete has been widely used for manufacturing sleepers in the world [2], and various attempts have been carried out to complement the brittle nature of the material. Cracks in concrete sleepers have been widely investigated and identified that it is mainly attributed to the material brittleness, particularly under dynamic loadings [1,3]. Although the tensile crack development in concrete is inevitable, it is revealed that the crack propagation can be effectively controlled by using various types of discontinuous reinforcements such as steel fibers [4]. Similarly, various efforts have also been made for concrete sleeper applications to enhance material ductility using fibers [5,6,7,8]. For example, Ramezanianpour et al. [5] used polypropylene fiber to enhance the tensile and flexural strength, and the durability of concrete by reducing chloride diffusion, water penetration, and sorptivity. Shin et al. [6] concluded that the use of 0.75% of steel fibers results in enhanced static and impact flexural capacity and toughness. Yang et al. [7] revealed that the concrete sleepers reinforced with steel fibers showed increased flexural and fatigue capacity at the rail-seat section compared with conventional concrete sleepers with conventional stirrups, since the concrete sleepers with steel fibers can mitigate crack propagation and prevent brittle shear failure. 

For an efficient massive concrete sleeper production process in a precast concrete facility, high strength concretes are generally used in order to promote early demolding and applying prestressing forces. Therefore, many national and international standards require specified minimum compressive strength of concrete for sleeper applications, e.g., C45/55 MPa in European standards [9], 50 MPa in Australia [10], and C50/60 MPa in the International Union of Railways recommendation [11]. Ultra high-performance concrete (UHPC) is one of the most advanced cement-based materials showing a compressive strength at 28 days higher than 150 MPa [12], which possesses strong potentials to extend the service lives of structures with various engineering merits including high ductility [13], durability [14], abrasion resistance [15], and impact resistance [16]. Recently, the authors revealed that the adoption of UHPC in railway sleepers resulted in stable structural behavior and outstanding crack resistance capability even after initial cracks developed [17].

Wide-width concrete sleepers are one of the special types of sleepers, which can significantly reduce the burden of ballast and track substructures due to a larger contact area. The large contact areas of the wide sleepers enable to reduce vibration values, extends maintenance intervals, and the life of the track system [18,19]. With these advantages, the range of applications of the wide concrete sleepers is getting higher from general lines to highly loaded areas such as transitional zones between earthwork, bridges, and tunnels.

Recently, there have been great efforts to study the numerical models of reinforced concrete structures by considering the nonlinearity of concrete behavior, bond strength, the stochastic natures of concrete, etc. Sucharda et al. [20] presented the nonlinear behavior of reinforced concrete beams without shear reinforcement using a stochastic model. In their concrete model, they incorporated the uncertainties in the concrete properties and studied the sensitivity to input parameters including fracture energy, *G_f_*. Instead of performing a direct tension test on concrete, they conducted splitting, three-point bending, and four-point bending tests. In their study, they reported that the ratio of the maximum to minimum loads is not necessarily corresponding to the limit of the input parameters. Valikhani et al. [21] studied numerical modeling of bonding of regular concrete and UHPC since UHPC can be used for the repair of concrete structures. In order to model the interface between concrete and UHPC, they used a zero-thickness volume element with post-failure tension-separation laws. They demonstrated the importance of the interface between two different materials. Shin and Yu [22] presented a numerical study on the splitting performance of prestressed concrete prisms by incorporating bond-slip behavior of prestressed concrete using a cohesive element. They used a user-defined material model to describe the bond-slip behavior at the interface. 

In this research, a numerical model of wide-type UHPC sleepers with respect to different amounts of fiber contents are developed and compared to the experimental tests. A direct tension test is performed and used for obtaining nonlinear properties of UHPC in tension. Using the developed models, a parametric study is performed to investigate the structural performance of the sleepers with respect to the content of steel fibers, the diameter of the prestressing tendons, and the yielding strength of PS tendons. The commercial finite element program ABAQUS is used in this study [23]. 

## 2. Mix Design and Fabrication of UHPC Sleeper

**Figure 1:**
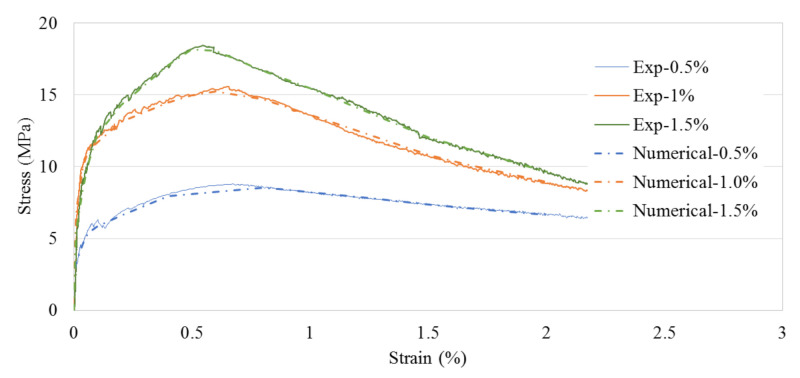
Experimentally obtained averaged stress-strain relationships under the direct tensile test on the UHPC with various fiber volume contents and the corresponding numerical constitutive models.

Wide-width concrete sleepers were manufactured using UHPC mixtures with three levels of fiber contents, mainly 0.5%, 1.0%, and 1.5%. The detailed mix proportion of the UHPC mixtures and the mixing procedures can be found in Bae and Pyo [8]. The compressive strength was evaluated using a 50 mm cubic specimen and averaged from at least three specimens. The compressive strengths of 0.5%, 1.0%, and 1.5%-UHPC specimens at 28 days were 149, 160, and 159 MPa, respectively. In addition, tensile strength results were adopted from Pyo et al. [24], where the tensile behavior of the similar UHPC mixture without ground granulated blast furnace slag (GGBFS) was characterized by following the JSCE recommendation [25]. The thickness of the tested tensile specimens was 30 mm-thick according to the recommendation. Figure 1 shows the averaged stress-strain relationships of UHPC with three levels of fiber contents under the direct tension test. For numerical constitutive models, the experimentally obtained constitutive relationship data were used to calibrate the uniaxial tensile behavior of three different concrete models. The solid lines represent the experimental data [24] and the dashed lines the numerical models. 

**Figure 2:**
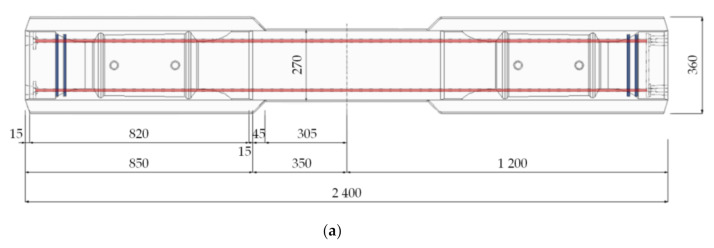

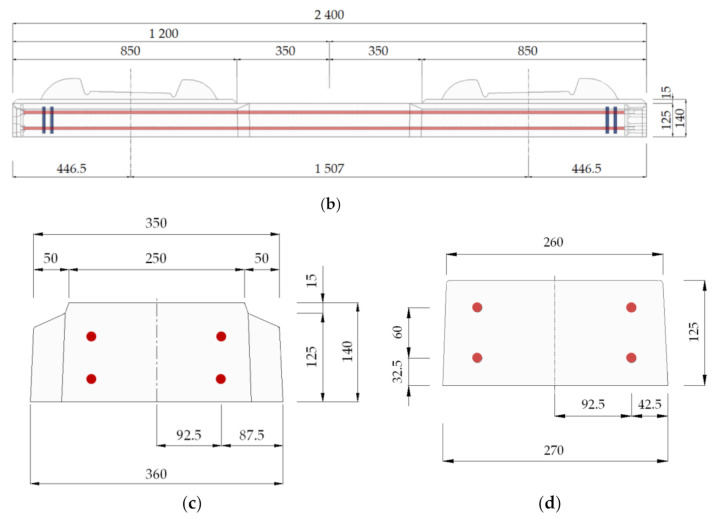
Geometrical dimension of L-150 series sleeper (unit: mm): (**a**) top view; (**b**) front view; (**c**) rail-seat section; (**d**) center section.

Figure 2 shows the detailed layout of the fabricated UHPC sleepers in the previous research [8,17], in which four PS tendons with diameters of 9.2 mm were used. Six sleepers with the 1.0% fiber volume case and three sleepers with the 0.5% and 1.5% fiber volume cases each were fabricated and tested. The fresh UHPC mixture was cast in the mold with external vibration, similar to the conventional sleeper production protocol. The casted UHPC sleepers were demolded after 24 h of curing and the sleepers were air-cured for an additional 24 h. Then, the prestress forces were introduced with the post-tensioning method. It is important to note that the prestress force was introduced without a post-tension duct and a thin layer of coating was applied to the surface of the PS tendons. 

## 3. Finite Element Modeling

The brittle cracking model available in ABAQUS [23] is adopted to describe the brittle failure nature of the concrete. Since the direct tensile stress and strain relationships were available for three different levels of the steel fiber contents, the direct stress after cracking and direct cracking strain data were computed and used to define concrete cracking behaviors. Figure 1 shows the comparisons between the numerical and experimental stress-strain relationships for the cracking models. The inelastic tensile strain is computed by Equation (1).

(1) $$\epsilon_{t}^{in}=\epsilon_{t}^{current}-\frac{\sigma_{t}^{current}}{E_{o}}\;,$$

where $\epsilon_{t}^{in}$ is the inelastic strain (direct cracking strain) in tension, $\epsilon_{t}^{current}$ is the total strain, $\sigma_{t}^{current}$ is the current stress level (direct stress after cracking), and $E_{o}\;$ is the initial elastic modulus of concrete [23]. For the simplicity and the elastic nature of the UHPC (with the compressive strength of 150 MPa), the compression region of concrete is modeled as a linear elastic model. Table 1 summarizes the important mechanical properties of concrete and prestresssing tendon in the models. For the post-cracking behavior of the UHPC, the direct stress onset of cracking was found to be 3.17 MPa, 6.52 MPa, and 5.58 MPa for the UHPC with steel fiber content 0.5%, 1.0%, and 1.5%, respectively, from the direct tension test. It is important to note that the direct stress onset of cracking of the UHPC with 1.0% steel fiber content is slightly higher than that of the UPHC with 1.5% steel fiber content. However, the ultimate strength of the UHPC with 1.5% steel fiber content is the highest (see Figure 1). 

**Figure 3:**
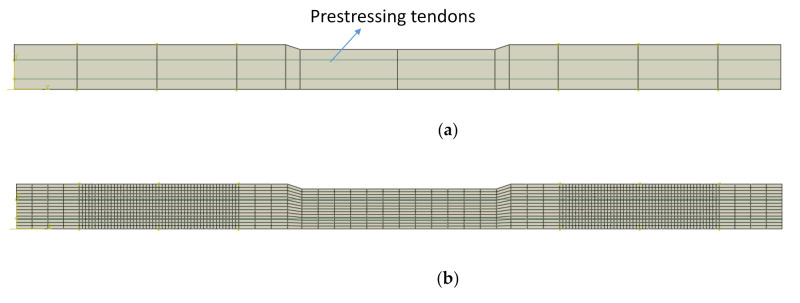

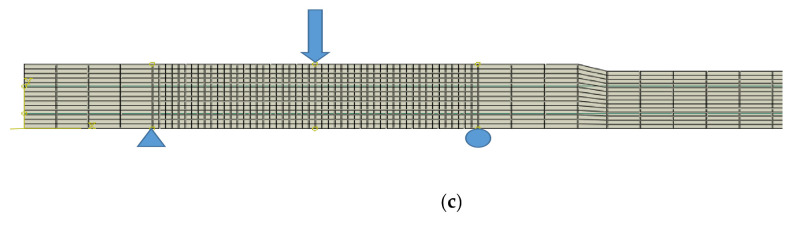
The 2D numerical sleeper model (**a**), its mesh (**b**), and the boundary conditions (**c**).

In this study, a 2D model with plane stress elements (four-node plane stress element) was adopted to describe the concrete body of a sleeper. The width of the sleeper is separately defined at the various regions; the width of 360 mm was assigned to the rail-seat area, and the width of 270 mm at the center section. The PS tendons were modeled as 1D truss model (two-node linear truss element) with a specific area (132.95 mm^2^ = 2 × 66.48 mm^2^) at the specific heights. The prestressing tendons were fully embedded into the concrete body. Figure 3 shows the 2D numerical model developed in ABAQUS (ABAQUS 6.14, Dassault Systèmes Simulia Corp, Providence, RI, USA). The total number of the elements and the nodes were 1840 and 1985, respectively. Then, 69,000 N (1038 MPa) of the prestressing force was assigned to each tendon. A pin and roller boundary conditions were assigned at 197 mm and 697 mm nodes from the free end. A point load was applied at 447 mm from the free end on the top surface at the rail-seat section, similar to the experimental test. An explicit dynamics analysis was performed for a quasi-static process [23]. 

## 4. Comparisons with Experimental Data

### 4.1. Summary of the Testing at the Rail-Seat Section

**Figure 4:**
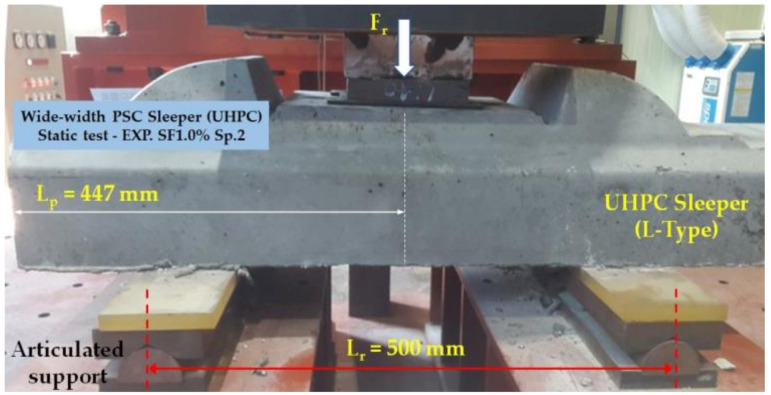
Static bending test setup at the rail-seat section.

A quasi-static three-point bending test according to European standards [9], was conducted on three UHPC sleepers with 0.5% steel fiber contents, six sleepers with 1.0% steel fiber contents, and three sleepers with 1.5% steel fiber contents. The centerline of the actuator is placed at 447 mm away from the free end of the sleeper, and the supports were placed 500 mm away from each other. Figure 4 shows a testing setup of the static three-point bending test. The reference test load, Fr_o_ of 126.8 kN, was computed [17]. The force and crack-width relationship of each sleeper was obtained and compared to each other. Overall, the higher the steel fiber contents are, the higher load capacities become. The 1.5% UHPC sleepers showed the highest failure forces and were able to mitigate the crack propagation. In Section 4.2.2, the experimental force and crack-width relationships together with numerical results are presented. The detail of the experimental tests and results can be found in the previous study [8].

### 4.2. Validation of the Numerical Sleeper Models

#### 4.2.1. Fiber Orientation Reduction Factor

**Figure 5:**
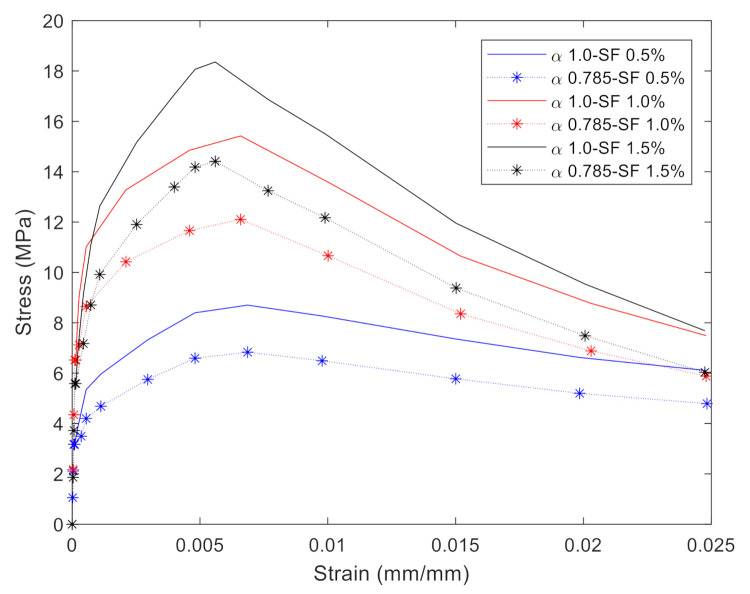
Stress and strain curves after applying the reduction factor of 0.785 for tension.

The fiber orientation effect was considered, herein, when calibrating the experimentally obtained tensile constitutive relationships of UHPC depicted in Figure 1. It is well known that the tensile capacities of fiber reinforced concrete including UHPC principally depends on the fiber properties including the distribution and volume fraction [26,27]. It should be pointed out that due to the relatively thin specimen used in the tensile test (30 mm) [24], 19.5 mm long steel fibers tend to be aligned in a two-dimensional manner. On the other hand, the discontinuous steel fibers can be assumed to be three-dimensionally oriented in the 140 mm thick rail-seat section of the sleepers. The fiber orientation factors, α, are known to be 2/π and 0.5 for two-dimensional and three-dimensional fiber orientations, respectively [27,28]. Therefore, it is logical to adopt 0.785 (=$\frac{0.5}{2/\pi }$) as the fiber orientation reduction factor in this numerical study. Figure 5 shows the adopted stress-strain curves in the numerical analysis after considering 0.785 of the reduction factor, α. After the proportional limit of the obtained stress-strain relationships, the strength is reduced by 21.5% of the original strengths. Therefore, the constitutive relationships with α of 0.785 were used in the numerical simulations.

#### 4.2.2. Validation of the Numerical Models

**Figure 6:**
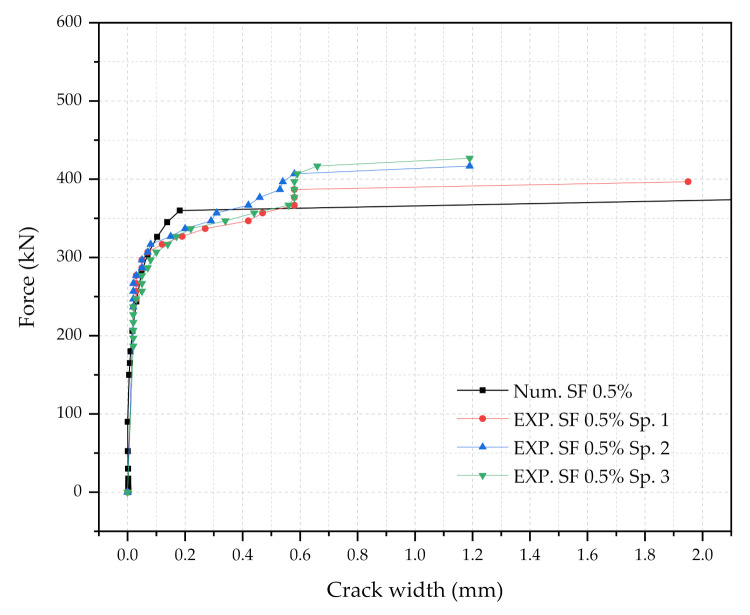
Comparison of the force and crack-width curves with 0.5% steel fiber UHPC at the rail-seat section.

**Figure 7:**
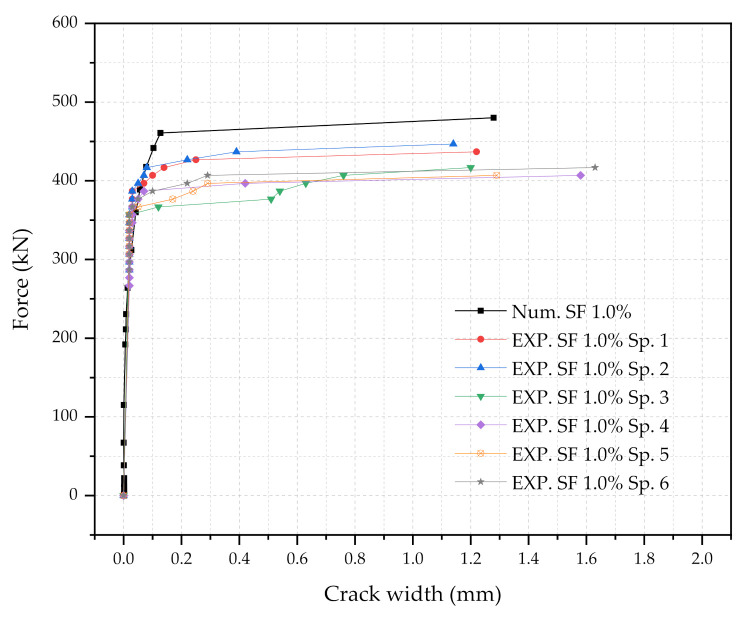
Comparison of the force and crack-width curves with 1.0% steel fiber UHPC at the rail-seat section.

**Figure 8:**
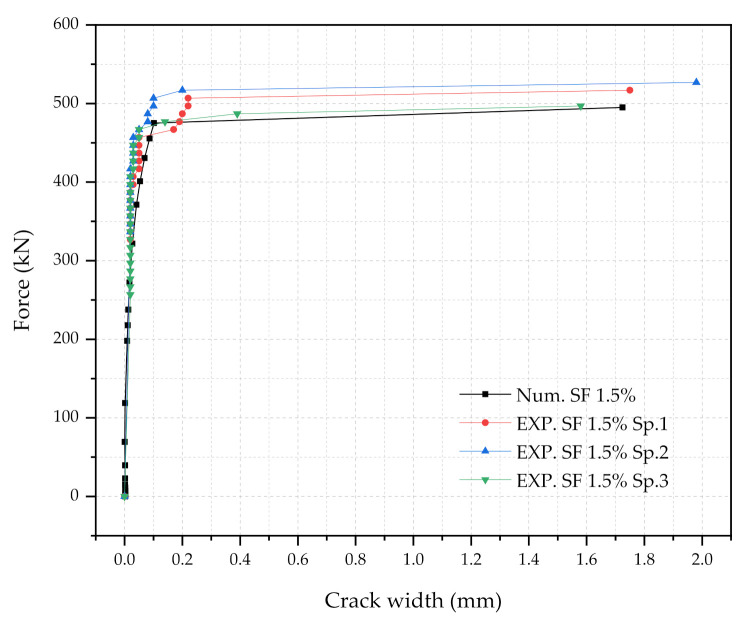
Comparison of the force and crack-width curves with 1.5% steel fiber UHPC at the rail-seat section.

Three numerical sleeper models were prepared: (1) with 0.5% steel fiber contents, (2) with 1.0% steel fiber contents, and (3) with 1.5% steel fiber contents. A point load is applied at the center of the rail-seat section of the models. The applied load and the crack widths were monitored and compared with the experimental tests. Figure 6, Figure 7 and Figure 8 show the comparisons of the force and crack-width curves between the numerical simulations and the experimental tests. Overall, the numerical models agree well with the experimental test results. The figures demonstrate that the sleeper models incorporated in this study are capable of capturing the initial stiffness, yielding of the steel tendons, cracking of the concrete, and the capacity of the sleepers due to the different level of steel fiber contents. It is also worthwhile to note that the fiber orientation factor, α, of 0.785 is able to describe the strength change in the UHPC of the sleepers from the coupon tests. In addition, the 1% steel fiber UHPC sleeper tends to overestimate the strength, while 0.5% and 1.5% steel fiber UHPC sleepers underestimate the ultimate strengths as compared to the experimental results.

## 5. Parametric Study

### 5.1. Design of Input Parameters

A parametric study was conducted with respect to the cross-sectional dimensions of sleepers and different types of steel tendons and steel fiber contents in the UHPC using the developed numerical sleeper models. In this parametric study, the structural performance of the UHPC sleepers were explored in terms of the crack width, the load capacities, the safety factor, and an economical design factor. 

**Figure 9:**
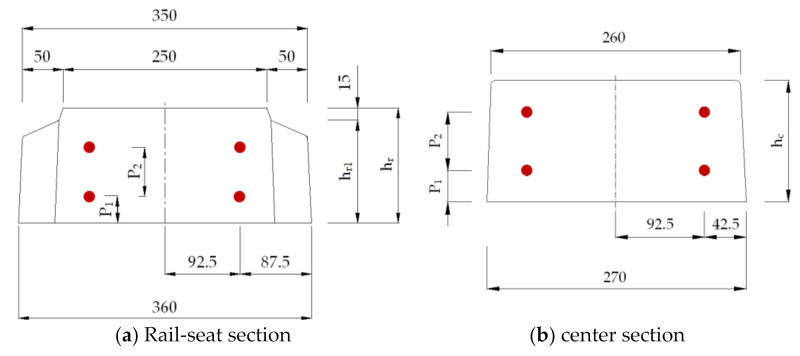
The schematics of the UHPC sleeper sections.

The important mechanical and geometrical parameters of the UHPC sleepers considered, herein, are as follows: (1) the cross-sectional dimensions, (2) the diameter of the steel tendons, (3) the yielding strength of the PS tendons, and (4) the steel fiber content of the UHPC. Table 2 summarizes the input values of each parameter. When the height of the cross-section at the rail-seat (h_r_) changes, the height of the cross-section (h_c_) changes accordingly. In addition, the locations of the steel tendons on top (P_1_) and bottom (P_2_) have to be adjusted (see Figure 9). Three different heights at the rail-seat section have been explored: 140 mm (L-type), 165 mm (M-type), and 195 mm (H-type). L, M, and H stands for lower, medium, and high height of the cross-sections, respectively. In the railway industry in South Korea, a 9.2 mm diameter tendon with 1080 MPa of the yielding strength has been commonly used. However, there is a growing interest in adopting larger diameter tendons and/or high strength steel such as 11.0 mm and 1275 MPa of the yielding strength when manufacturing prestressed concrete sleepers. Three different levels of steel fiber contents (0.5%, 1.0%, and 1.5%) are also explored. The total number of the simulation cases is 21, and Table 3 summarizes the 21 different simulation cases. Specimen numbers 1~7 were designed to have 0.5% of steel fiber of the UHPC, specimen numbers 8~14, 1.0%, and specimen numbers 15~21, 1.5%, respectively.

### 5.2. Analysis Results

#### 5.2.1. Cross-Sectional Dimensions: L, M and H

**Figure 10:**
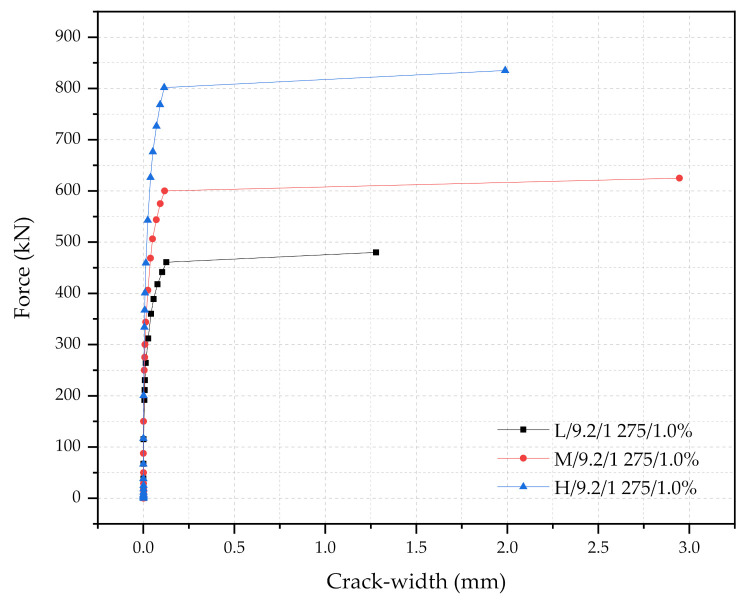
The force-crack width diagram of the L, M, and L-type sleepers with 9.2 mm diameter, 1% steel fiber, and fy of 1275 MPa.

In order to discuss the effect of the cross-section sizes (L, M, and H), three simulation results were presented in Table 4 and Figure 10 as examples: (1) L/9.2/1275/1.0%, (2) M/9.2/1275/1.0%, and (3) H/9.2/1275/1.0%. In this discussion, the only variable is the size of the cross-section, when other parameters are kept constant: the diameter of the tendons is 9.2 mm, the yielding strength of the tendon is 1275 MPa, and the steel fiber content is 1.0%. In general, the larger the cross-section is, the greater the loading capacity of the sleepers becomes. In the figure, the simulation result of the sleeper with 140 mm of h_r_, 9.2 mm of the diameter, 1275 MPa of f_y_ (steel), and 1% of the steel fiber content is represented by the black square line (L/9.2/1275/1%). ΔF_1_ means the change in the applied load required between the force (Fr_r_) when the crack width is about 0.01 mm and the corresponding force (Fr_0.05_) when the crack width reaches about 0.05 mm. Higher ΔF_1_ is observed from the larger section sleepers. This means that the larger cross-section sleepers are capable of delaying crack propagations. In other words, when the cross-section of the sleeper gets larger, the moment of inertia becomes greater, which results in increased flexural rigidity and sustains higher moments without significant damages. After the crack width reached 0.05 mm, the secant and tangent modulus of the force-crack width diagram were gradually reduced. At approximately 0.12 mm crack width, the PS tendons reached the yielding. Soon after the yielding of the prestressing tendons, the sleepers reached the failure (Fr_B_) of the rail-seat section due to the significantly reduced flexural rigidity. Similar trends were observed when the steel fibers were 0.5% and 1.5% as well. The force and crack-width graphs of other cases are presented in Section 5.2.3. 

The ratio of the cross-sectional area of the M-type sleeper to the L-type sleeper, and the ratio of the cross-sectional area of the H-type sleeper to the L sleeper are 1.19 and 1.41, respectively, while the ΔF_1_ ratios of the M to L sleeper and H to L sleeper were 1.30 and 1.74, respectively. This means that the increased capacity ratios of the sleepers were higher than the increased area ratios. The safety factor of each sleeper can be computed by $\frac{F_{rB}}{2.5F_{ro}}$, where Fr_B_ and Fr_o_ is the force at the failure and the design reference force; Fr_o_ is 126.8 kN and 2.5 is the dynamic factor [17]. L, M, and H’s safety index was found to be 1.51, 1.97, and 2.64. Too large a safety index means the sleeper is over-designed. This study suggests an economical design factor, which can be computed by 100Fr_B_/Area. When this index is close to 1, the sleeper is structurally sound and economical. The 100Fr_B_/Area index value of the L, M, and H sleepers were found to be 1.02, 1.12, and 1.25, which indicate that the L-type sleeper is the most economical design.

#### 5.2.2. The Diameter and the Yielding Strength of PS Tendons

**Figure 11:**
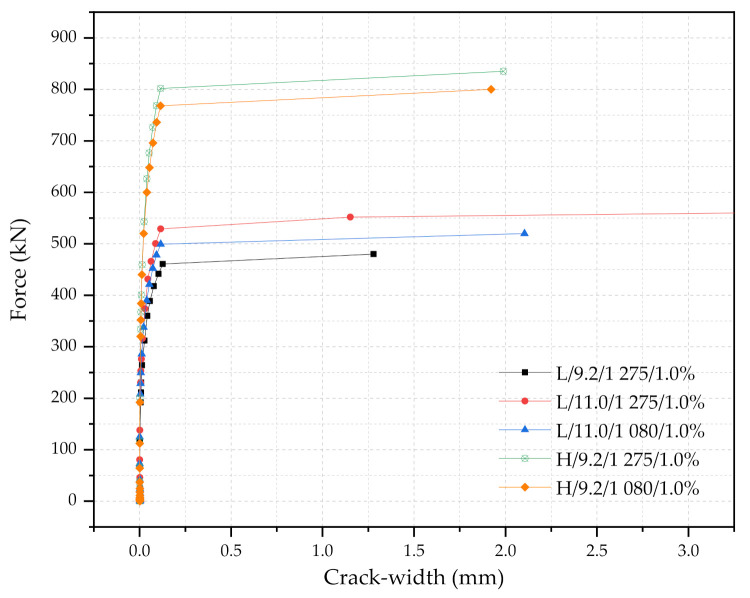
Force and crack width diagrams with respect to the diameter and the yielding strength of the PS tendons (the steel fiber content was kept at a 1.0% constant).

Table 5 and Figure 11 shows the simulation results with respect to the diameter and the yielding strength of the PS tendons when the steel fiber content was kept at 1.0%. Two different diameters of the PS tendons are explored: (1) 9.2 mm (smaller diameter), and (2) 11.0 mm (larger diameter). In addition, 1080 MPa and 1275 MPa of the yielding strength, f_y_ are considered. As examples, five simulations are presented in Table 5 and Figure 11: (1) L/9.2/1275/1.0%, (2) L/11.0/1275/1.0%, (3) L/11.0/1080/1.0%, (4) H/9.2/1275/1.0%, and (5) H/9.2/1080/1.0%. Given that the cross-sections and the steel fiber contents are kept constant, about 20% higher yielding strength PS tendons results in only 4.4% and 9.5% increase in ΔF_2_ for H/9.2 types, and L/11.0 types, respectively. This is due to the area of the PS tendons to the area of the cross-sectional area of concrete being relatively low for the H/9.2 type. When using the larger diameter PS tendons, the load capacities of the sleepers increase accordingly. When comparing the results between L/9.2/1275/1.0% and L/11.0/1275/1.0%, the area of the larger diameter PS tendons is 1.43 times to that of the smaller diameter tendons; and the increase in Fr_r_, Fr_0.05_, and Fr_B_ is 20%, 11%, and 20%, respectively. These results indicate that the use of the larger diameter tendons would be more efficient than the use of the higher strength PS tendons in terms of the load increase capacities. In addition, these simulation results give some insights on whether sleeper (or crosstie) engineers would like to use a combination of (1) smaller diameter with higher strength PS tendons or (2) larger diameter with lower strength PS tendons. L/11.0/1080/1.0% case shows higher load capacities and safety factors than those from L/9.2/1275/1.0%. However, when engineers prefer an economical design, L/9.2/1275/1.0% can also be adopted since the safety factor is 1.51 and the 100Fr_B_/Area index is close to 1.0. 

#### 5.2.3. Steel Fiber Contents

**Figure 12:**
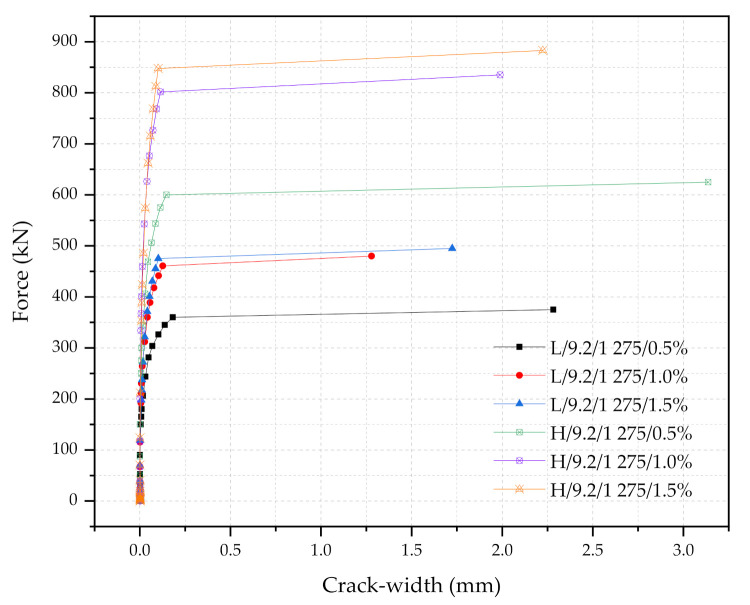
Force and crack-width relationships of the L- and H-type sleepers with respect to the three different steel fiber contents.

This section presents the simulation results with respect to the steel fiber contents (i.e., 0.5%, 1.0%, and 1.5%). Table 6 and Figure 12 show the summary of the results of the six simulations used as examples: (1) L/9.2/1275/0.5%, (2) L/9.2/1275/1.0%, (3) L/9.2/1275/1.5%, (4) H/9.2/1275/0.5%, (5) H/9.2/1275/1.0%, (6) H/9.2/1275/1.5%. In general, as the steel fiber content increases, the load capacities, the safety factor, and the economic design factor increase. For the smaller cross-section sleepers (L-type cases), the use of 1.0% and 1.5% steel fiber contents results in the significant increase in the performance compared to that of the sleeper with 0.5% steel fiber content. The performance of L/9.2/1275/1.0% and L/9.2/1275/1.5% are similar to each other, and the increase in the load capacities are only 3~6%; furthermore, the safety factor only increases to 1.56 (1.5% of the steel fiber) from 1.51 (1.0% of the steel fiber). The performance of the L-type-0.5% steel fiber sleeper is significantly lower than that of the sleepers with the higher steel fiber contents. As observed, $\frac{F_{rB}}{2.5F_{ro}}$ is only 1.18 and 100Fr_B_/Area is 0.79 for L/9.2/1275/0.5%. For the larger cross-section sleepers (H-type cases), the trends are similar to those from the L-type cases. The steel fiber 1.0% and 1.5% sleepers show good performance while the difference between two cases is not as great as the L-types. The H-type-0.5% steel fiber sleeper shows lower load capacities and safety factors when compared to those of the higher steel fiber content sleepers; 100Fr_B_/Area is 0.94, which is still less than 1.0. When casting a smaller cross-section UHPC sleeper (L-type case), the use of 0.5% steel fiber content is not adequate. In addition, the difference in the performance of the sleepers between 1.0 and 1.5% steel fiber UHPC in terms of the force and crack-width at the rail-seat is not much different. Therefore, 1.0% steel fiber UHPC can be an economical design choice. For the larger cross-section sleepers, all three steel fiber contents would be acceptable. However, instead of H-type 0.5% sleeper, M-type sleepers could be a good alternative since M-type sleepers shows the similar performance while they are more economical. As an example, the performance of M/9.2/1275/1.0% is similar to that of H/9.2/1275/0.5% in term of Fr_r_, Fr_0.05,_ Fr_B,_ the economical design factor, and the safety factor (see Table 4 and Table 6). The economical design factor, 100Fr_B_/Area of the M-type was found to be 1.12, while that of the H-type was 0.94.

**Figure 13:**
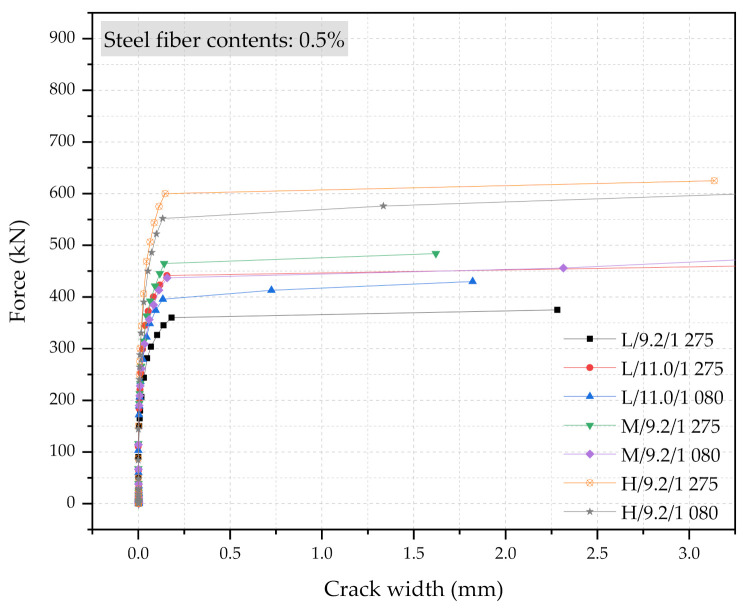
Simulation results of the force and crack-width curves with 0.5% steel fiber UHPC at the rail-seat section.

**Figure 14:**
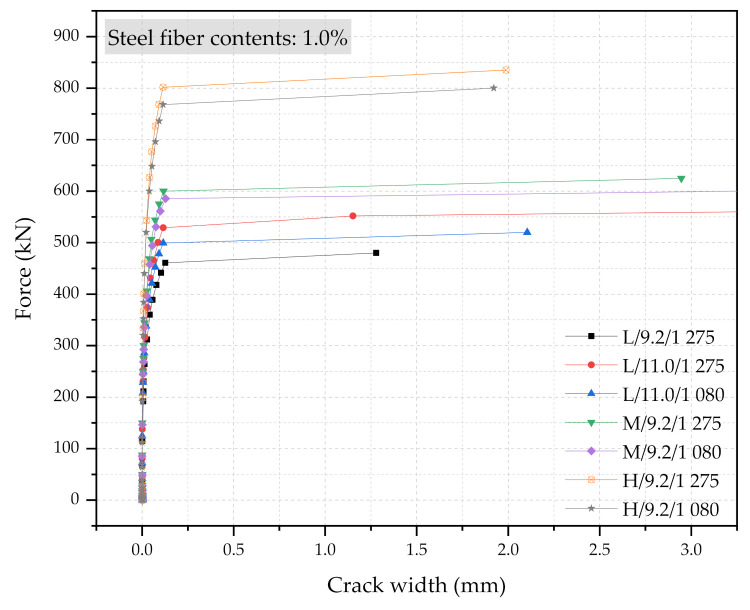
Simulation results of the force and crack-width curves with 1.0% steel fiber UHPC at the rail-seat section.

**Figure 15:**
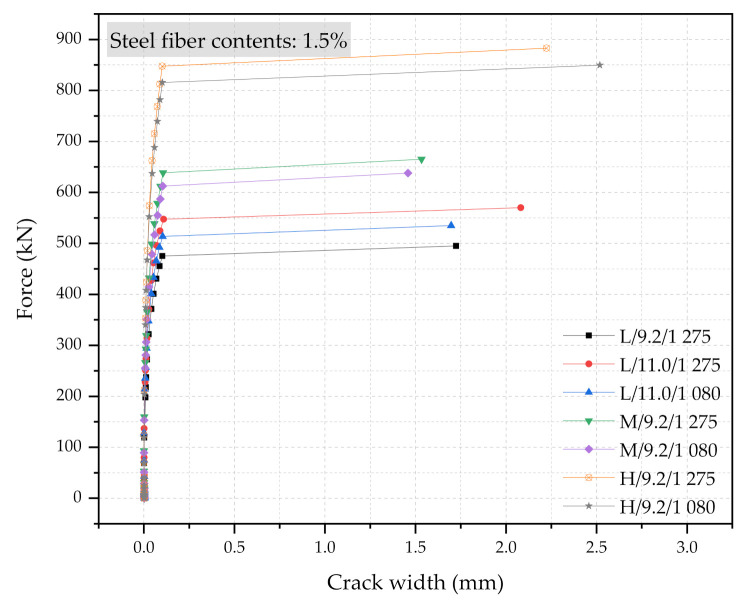
Simulation results of the force and crack-width curves with 1.5% steel fiber UHPC at the rail-seat section.

Figure 13, Figure 14 and Figure 15 show the force and crack-width relationship of all 21 numerical simulations. Regardless of the steel fiber contents, the increase in size would have the most significant impacts on the performance of the UHPC sleepers. The improvement can be further enhanced with the combination of the higher steel fiber contents, the larger diameter PS tendons, and the higher strength PS tendons. However, the use of a larger cross-section with 1.5% steel content, 11.0 mm diameter PS tendons of 1275 MPa yielding strength is a clearly over-designed sleeper. This study also indicates that the improvement due to the higher strength PS tendons would be minimum among all the design parameters considered. It is also interesting to note that there are cases that would show similar performance even though the cross-section sizes are different. For example, M/9.2/1080/0.5% and L/11.0/1275/0.5% show similar performance, as well as M/9.2/1275/1.0% and H/9.2/1275/0.5%.

## 6. Discussion

In this numerical study, a 2D prestressed concrete sleeper model with a brittle cracking constitutive model was developed and validated with experimental data. In general, the numerical results were compatible with the experimental results in terms of the force and crack-width relationship at the rail-seat section. The obtained tensile stress-strain relationships of UHPC with different steel fiber contents [24] were directly used to define the cracking stress and cracking strain of the brittle cracking model. In this process, an orientation reduction factor of 0.785 was applied to the post-cracking behavior of all three UHPCs. As it has been known to the community, 2D and 3D orientation reduction factors are 2/π, and 0.5. Therefore, a single orientation reduction factor (0.785) was adopted in this study for the simulations. However, the fiber reduction factor could be dependent upon the distribution, orientation, and volume fraction of the fibers in a concrete mix. Therefore, it is challenging to use a just deterministic approach for the factor while the concrete properties including the reduction factor has the stochastic characters. With the 0.785 reduction factor, 1.0% steel fiber sleepers predict the ultimate strength higher than the experimental test. This shows that there is room for improvement. A stochastic approach can be applied to evaluate the range in the performance of the sleepers using UHPC. It should be, however, pointed out that additional experimental research should be conducted to achieve a statistically meaningful dataset to adopt the stochastic approach, especially for the fiber orientation effect. In addition, the usage of a single orientation reduction factor would be beneficial for sleeper engineers and structural engineers to practically design concrete structures with fiber reinforcements.

## 7. Conclusions

This numerical study focuses on investigating the performance of UHPC sleepers with respect to various design/mechanical/geometrical parameters. The parameters include the steel fiber contents, the size of the cross-section, the diameter and yielding strength of the PS tendons. The key observations and findings of this research can be summarized as follows:

1. The developed numerical 2D-UHPC sleeper model was capable of representing the force and crack-width relationships. Three UHPC direct tension tests with the 0.5%, 1.0%, and 1.5% steel fiber contents were used for the UHPC tensile constitutive models.
2. The fiber orientation factor, α, of 0.785 is used to represent the realistic stress-strain behavior of the UHPC in 3D as opposed to the thin coupon test where the fibers are well aligned in a 2D manner.
3. The numerical analysis results indicate that the bigger the cross-section is, the higher the load capacities and the safety factor become. However, using a too large cross-section can result in uneconomical design sleepers. The economical design factor, 100Fr_B_/Area is computed to evaluate the economical factor of the UHPC sleeper. When 100Fr_B_/Area is close to 1.0, the UHPC sleeper is economical.
4. There are growing interests in using a larger diameter tendon and/or a higher strength tendon. This study recommends using a larger diameter tendon with a lower strength for an economical design.
5. A steel fiber content of 0.5% tends to yield to lower strengths UHPC sleepers relative to the 1.0% and 1.5% steel fiber content sleepers.
6. Some M-type sleepers with 1.0% steel fiber UHPC show similar performance to H-type sleepers with 0.5% steel fiber UHPC.

This numerical study was able to provide insights on the effects of the design parameters for developing concrete sleepers using UHPC. Additional research needs to be conducted to investigate the overall behavior of UHPC sleepers, including bending at the center-section of the sleepers and the effect of the variability of concrete properties including an orientation reduction factor. 

## Figures and Tables

**Table 1:** Summary of the material properties.

**Concrete**	Young’s modulus	51.0 GPa
	Compressive strength	150 MPa
	Poisson’s ratio	0.2
	Tensile strength (steel fiber 0.5%)	8.82 MPa
	Tensile strength (steel fiber 1.0%)	15.6 MPa
	Tensile strength (steel fiber 1.5%)	18.4 MPa
	Direct stress onset of cracking (steel fiber 0.5%)	3.17 MPa
	Direct stress onset of cracking (steel fiber 0.5%)	6.52 MPa
	Direct stress onset of cracking (steel fiber 0.5%)	5.58 MPa
**Steel tendon**	Young’s modulus	200 GPa
	Yielding strength	1275 MPa
	Poisson’s ratio	0.3

**Table 2:** Summary of the input parameters.

Steel fiber contents (%)	0.5	1.0	1.5
Yielding stress of prestressing tendon (f_y_) (MPa)	1 080	1 275	
Diameter of prestressing tendon (φ) (mm)	9.2	11.0	
Cross-sectional parameters	L-Type	M-Type	H-Type
Height of the rail-seat section, h_r_ (mm) (h_r1_, mm)	140 (125)	165 (150)	195 (180)
Height of the center section, h_c_ (mm)	125	150	180
Location of the prestressing tendon, P_1_ (mm)	32.5	35	50
Location of the prestressing tendon, P_2_ (mm)	60	75	80

**Table 3:** Summary of the numerical concrete sleeper models and their nomenclatures.

Sp. No.	Steel Fiber 0.5%	Sp. No.	Steel Fiber 1.0%	Sp. No.	Steel Fiber 1.5%
No.1	L-φ9.2-f_y_1 275	No.8	L-φ9.2-f_y_1 275	No.15	L-φ9.2-f_y_1 275
No.2	L-φ11.0-f_y_1 275	No9	L-φ11.0-f_y_1 275	No.16	L-φ11.0-f_y_1 275
No.3	L-φ11.0-f_y_1 080	No.10	L-φ11.0-f_y_1 080	No.17	L-φ11.0-f_y_1 080
No.4	M-φ09.2-f_y_1 275	No.11	M-φ9.2-f_y_1 275	No.18	M-φ9.2-f_y_1 275
No.5	M-φ09.2-f_y_1 080	No.12	M-φ9.2-f_y_1 080	No.19	M-φ9.2-f_y_1 080
No.6	H-φ09.2-f_y_1 275	No.13	H-φ9.2-f_y_1 275	No.20	H-φ9.2-f_y_1 275
No.7	H-φ09.2-f_y_1 080	No.14	H-φ9.2-f_y_1 080	No.21	H-φ9.2-f_y_1 080

**Table 4:** Summary of the L, M, and H-type sleepers with the following parameters: 9.2 mm diameter, fy of 12,175 MPa and 1% steel fiber.

Simulation Case	Rail-Seat Section Area (mm^2^)	Force (kN)		Crack Width (mm)	100Fr_B_/Area	ΔF_1_ (kN) = (Fr_0.05_ − Fr_r_)	ΔF_2_ (kN) = (Fr_B_ − Fr_0.05_)	Fr_B_/2.5Fr_0_
L/9.2/1 275/1.0%	47 200	Fr_r_	230.4	0.008 6	1.02	158.4	91.2	1.51
		Fr_0.05_	388.8	0.056 4				
		Fr_B_	480.0	1.28				
M/9.2/1 275/1.0%	56 075	Fr_r_	300.0	0.009	1.12	206.2	118.8	1.97
		Fr_0.05_	506.2	0.051				
		Fr_B_	625.0	2.94				
H/9.2/1 275/1.0%	66 725	Fr_r_	400.8	0.009 4	1.25	276.5	158.7	2.63
		Fr_0.05_	676.3	0.052 8				
		Fr_B_	835.0	1.988				

**Table 5:** Summary of the simulation results with respect to the diameter and the yielding strength of the PS tendons (the steel fiber content was kept at a 1.0% constant).

Analysis Case	Rail-Seat Section Area (mm^2^)	Force (kN)		Crack Width (mm)	100Fr_B_/Area	ΔF_1_ (kN) = (Fr_0.05_ − Fr_r_)	ΔF_2_ (kN) = (Fr_B_ − Fr_0.05_)	Fr_B_/2.5Fr_0_
L/9.2/1 275/1.0%	47 200	Fr_r_	230.4	0.0086	1.02	158.4	91.2	1.51
		Fr_0.05_	388.8	0.0564				
		Fr_B_	480.0	1.28				
L/11.0/1 275/1.0%	47 200	Fr_r_	276.0	0.0098	1.22	155.3	142.7	1.81
		Fr_0.05_	431.3	0.0452				
		Fr_B_	575.0	7.292				
L/11.0/1 080/1.0%	47 200	Fr_r_	249.6	0.0115	1.10	171.6	98.8	1.64
		Fr_0.05_	421.2	0.0532				
		Fr_B_	520.0	2.104				
H/9.2/1 275/1.0%	66 725	Fr_r_	400.8	0.0094	1.25	276.5	158.7	2.63
		Fr_0.05_	676.3	0.0528				
		Fr_B_	835.0	1.988				
H/9.2/1 080/1.0%	66 725	Fr_r_	384.0	0.0084	1.12	264.0	152.0	2.52
		Fr_0.05_	648.0	0.0539				
		Fr_B_	800.0	1.922				

**Table 6:** Summary of the simulation results with respect to the 0.5%, 1.0%, and 1.5% steel fiber contents.

Analysis Case	Rail-Seat Section Area (mm^2^)	Force (kN)		Crack Width (mm)	100Fr_B_/Area	ΔF_1_ (kN) = (Fr_0.05_ − Fr_r_)	ΔF_2_ (kN) = (Fr_B_ − Fr_0.05_)	Fr_B_/2.5Fr_0_
L/9.2/1 275/0.5%	47 200	Fr_r_	180.0	0.0103	0.79	101.3	93.7	1.18
		Fr_0.05_	281.3	0.0487				
		Fr_B_	375.0	2.282				
L/9.2/1 275/1.0%	47 200	Fr_r_	230.4	0.0086	1.02	158.4	91.2	1.51
		Fr_0.05_	388.8	0.0564				
		Fr_B_	480.0	1.28				
L/9.2/1 275/1.5%	47 200	Fr_r_	217.8	0.0108	1.05	183.1	94.1	1.56
		Fr_0.05_	400.9	0.0532				
		Fr_B_	495.0	1.724				
H/9.2/1 275/0.5%	66 725	Fr_r_	300.0	0.0105	0.94	168.7	156.3	1.97
		Fr_0.05_	468.7	0.0454				
		Fr_B_	625.0	3.137				
H/9.2/1 275/1.0%	66 725	Fr_r_	400.8	0.0094	1.25	276.5	158.7	2.63
		Fr_0.05_	676.3	0.0528				
		Fr_B_	835.0	1.988				
H/9.2/1 275/1.5%	66 725	Fr_r_	388.5	0.0109	1.32	273.8	220.7	2.79
		Fr_0.05_	662.3	0.0452				
		Fr_B_	883.0	2.224				

## Data Availability

The data presented in this study are available on request from the corresponding authors.
